# *Phoenix dactylifera* mediated green synthesis of Cu_2_O particles for arsenite uptake from water

**DOI:** 10.1080/14686996.2016.1244472

**Published:** 2016-11-16

**Authors:** Mokhtar Ali Amrani, Vadali V. S. S. Srikanth, Nitin K. Labhsetwar, Ahmed S. Al- Fatesh, Hamid Shaikh

**Affiliations:** ^a^ School of Engineering Sciences and Technology (SEST), University of Hyderabad, Hyderabad, India; ^b^ Environmental Materials Division, CSIR-National Environmental Engineering Research Institute (CSIR-NEERI), Nagpur, India; ^c^ Chemical Engineering Department, College of Engineering, King Saud University, Riyadh, Saudi Arabia

**Keywords:** *Phoenix dactylifera*, glucose, cuprous oxide, arsenic(III) ions, green synthesis, 10 Engineering and Structural materials, 100 Materials, 304 Powder processing / Sintering, 307 Kinetics and energy / mass transport, 501 Chemical analyses, 503 TEM, STEM, SEM

## Abstract

In this study, an environmentally friendly, cost-effective, and single-step procedure is used for the synthesis of polycrystalline Cu_2_O particles with controlled morphologies. Simple sugars are extracted from date fruit pulp (*Phoenix dactylifera*) and used as a reducing agent for the formation of Cu_2_O particles in aqueous medium. The feasibility of this solution is compared with the standard glucose solution. The Cu_2_O particles are characterized by electron microscopy, X-ray diffraction, optical absorption and Raman scattering techniques. It is concluded that the morphology of the particles is mainly influenced by the solvents. The obtained Cu_2_O particles are then used as an adsorbent to uptake As(III) ions from water. The maximum adsorption capacity (*Q*_max_) is estimated by Langmuir and Freundlich isotherms and it is found that *Q*_max_ = 14.3 mg g^–1^. Adsorption kinetics study showed that the adsorption equilibrium could be achieved in 1 h and that the purified water meets the standards of World Health Organization (WHO) for acceptable amount of As(III) in drinking water. Adsorption kinetic models showed that the adsorption is chemisorption in nature.

## Graphical abstract

**Figure:**
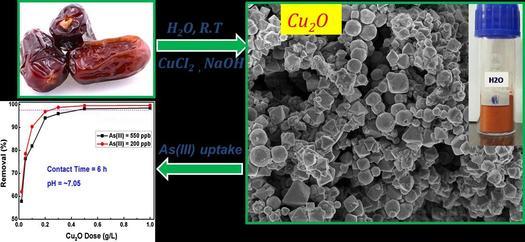

## 1. Introduction

Cuprous oxide (Cu_2_O), a non-stoichiometric p-type semiconducting material, has attracted much attention for its catalytic, antibacterial, energy and environmental applications.[1,2] It is also considered as a competent adsorbent for water purification due to its low toxicity and negligible solubility in water.[3–5] Therefore, great efforts have been devoted to the synthesis of cheap, clean and surface-engineered Cu_2_O particles in recent years.

Date fruit pulp is one of the most abundant natural sources of carbohydrate sugars. It contains about 70–90% of sugars in the form of glucose, sucrose, fructose and other reducing sugars. The major sugars are glucose and fructose.[6,7] Apart from their chemical fingerprints, date palm wastes have been widely used as conventional low-cost adsorbents for heavy metal uptake from wastewater.[8] It also contains a considerable amount of proteins, antioxidants such as ascorbic acids, amino acids, phenolics, flavonoids and minerals.[6,7] Therefore, this unique product can be explored to catalyze reactions as well as to make hydrophobic interactions with metallic ions in a suitable solvent. In this context, Cu_2_O has recently been synthesized with the aid of natural carbohydrate sources.[9–11] However, its synthesis with the aid of date fruit pulp extract has not yet been elucidated.

Arsenic contamination in water is a global problem since arsenic occurs naturally in the soil and water in high concentrations.[12] Efficient technologies are therefore needed to remove it from water. Uptake of ionic contaminants by means of adsorption is one of the most efficient methods owing to its ease, low expense and high selectivity. Various other adsorbents, derived from natural products or industrial wastes have been developed for the removal of arsenic from water.[8] However, most of the existing adsorbents have disadvantages such as inaccessibility, toxicity, solubility in water and time-consuming synthesis processes. Recently, metal oxides have been widely used as adsorbents for arsenic uptake from water.[13] Cu_2_O has never been used as an adsorbent to remove arsenic from water while CuO has been used.[14–16]

In addition to the limited synthesis methods of crystalline Cu_2_O particles, the prediction of simple, clean and cost-effective methods for the synthesis of pure and surface-engineered Cu_2_O particles is still a challenge. Herein, different characteristics of polycrystalline Cu_2_O particles synthesized with the aid of date fruit pulp extract are discussed. The synthesized Cu_2_O particles exhibited excellent adsorption characteristics and were used to remove As(III) from water.

## 2. Materials and experiments

### 2.1. Preparation of the extract solution

A good quality date fruit pulp was purchased from the local market, Riyadh, Saudi Arabia. Ten grams of the date fruit pulp were added to 100 ml boiled water (120 °C) for 10 min and then the resultant solution was vacuum filtered to remove the undissolved constituents. The solution turned a yellowish color, indicating high extraction. Note that extraction in alkaline solutions and/or organic solvents is preferred for higher extraction efficiency.

### 2.2. Synthesis of the Cu_2_O particles

In a typical synthesis method, 3.4 g of CuCl_2_ ⋅ 2H_2_O (Merck, India, 99%) and 3 g of NaOH pellets (Fisher Scientific, India, 98%) were dissolved in 100 ml distilled water each and properly mixed together followed by addition of 40 ml of date fruit pulp extract solution (DFPES) under vigorous stirring under room conditions for 1 h. During this process, the color of the solution changes gradually into a brick-red color, indicating the formation of cuprous oxide (Cu_2_O). The resultant solution was refluxed and allowed to precipitate; the precipitate was subsequently filtered and washed with ethanol, distilled water and acetone, in turn. The resultant precipitate was finally dried at 120 °C for 3 h to obtain a fine powder. The experiment was repeated with ethanol and ethylene glycol as the solvents (exclusively for the copper precursor) in place of distilled water.

### 2.3. Adsorption experiments

Adsorption experiments were performed to uptake the As(III) ions from water with the aid of Cu_2_O powder as the adsorbent. The powder was also dispersed in the arsenic water for predefined contact time and then filtered. Removal efficiency (*R*(%)) and adsorption capacity (*Q*_*e*_) in mg g^–1^ can be estimated by these equations:

(1) $$R\left(\%\right)=\frac{(C_{\text{0}}-C_{e})*100}{C_{\text{0}}}$$

(2) $$Q_{e}=\frac{(C_{\text{0}}-C_{e})*1000}{W/V}$$

where *C*_0_ and *C*_*e*_ are the As(III) concentrations in μg l^–1^ before and after the adsorption process, respectively. The values of *C*_*e*_ and *C*_0_ are obtained directly by inductively coupled plasma mass spectroscopy (ICP-MS). *W/V* is the Cu_2_O dose in g l^–1^. Equations (1) and (2) are used to plot the data and to estimate the adsorption parameters.

A dose study was performed at two different As(III) concentrations (200 and 550 ppb) and eight doses of Cu_2_O adsorbent in the range of 0.02 to 1 g l^–1^. As(III) solution was prepared by adding reagent grade As_2_O_3_ of 99.8% purity (Inorganic Ventures, Christiansburg, VA, USA) to 1000 ml of distilled water. Conical flasks of 250 ml capacity were used for the dose experiments. Each conical flask was filled with 100 ml of the As(III) solution and the corresponding Cu_2_O dose. Adsorption kinetics at different contact times (1–120 min) were studied for two different Cu_2_O doses (0.25 and 0.5 g l^–1^). The concentrations of As(III) ions in the water were measured at seven different contact times (1–120 min). In the final step, samples were filtered using 0.22 μm disposable membrane filters and the filtrated water was analyzed using ICP-MS (NexIon 300X, Perkin Elmer, UK).

## 3. Characterization

The morphology of the particles was studied using field emission scanning electron microscope (FESEM, Ultra 55 of Carl Zeiss, AG, Germany) operated at an accelerating voltage of 5 kV. The structure phase and crystallinity of the samples were studied by X-ray diffraction (XRD) patterns which were recorded from 20 to 80 ° using Cu Kα as the X-ray source (*λ* = 1.54 Å; Bruker’s AXS Model D8 Advance System, Germany). Fourier transform infrared (FTIR) spectroscopy (Nicolet 380, Thermo Scientific, USA) measurements were used to study the surface properties and functional groups in the samples. Raman spectroscopic study was performed to understand the phase characteristics and vibrational modes of the Cu_2_O atoms. It was carried out using 532 nm laser line of Nd-YAG laser with spectral resolution of 3 cm^−1^. UV-vis spectroscopy (V-570, JASCO, USA) was used to study the optical properties of the samples. It uses a resolution of 1 nm in the range of 200–800 nm at a scanning speed of 200 nm min^–1^.

## 4. Results and discussion

### 4.1. Structural and morphological studies

A facile, simple and straightforward synthesis pathway has been implemented for the synthesis of Cu_2_O particles with the aid of the DFPES. The reddish color of the as-prepared powder is the first indication of the formation of the Cu_2_O particles. The unique composition of the sugars present in DFPES may be the reason behind the effective and rapid reduction of copper ions to copper (I) oxide particles. This synthesis method can be scaled up for the gram scale production with mono-dispersed and stable particles. In fact, our laboratory experiments have resulted in synthesizing ~50 g in a single experiment.

**Figure 1.**
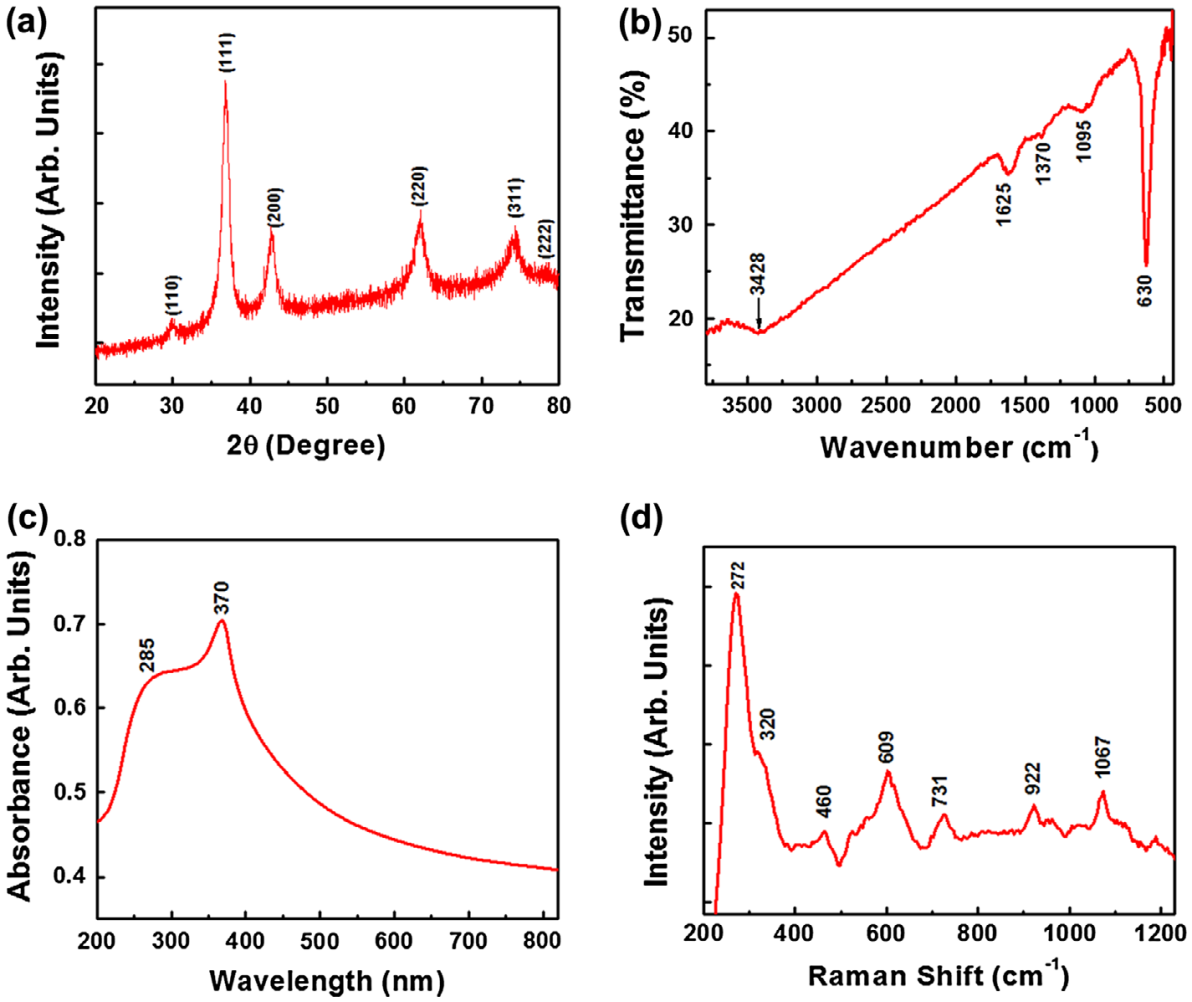
(a) X-ray diffractogram, (b) FTIR spectrum, (c) UV-vis absorption spectrum and (d) Raman spectrum of the Cu_2_O particles. DFPES is the reducing agent while water is the solvent.

Figure 1(a) shows X-ray diffractograms of the Cu_2_O powder, for the analysis of the crystallite size and phase purity. The typical XRD pattern of the Cu_2_O particles shows the Bragg’s reflections at ~29.9 °, ~36.9 °, ~42.6 °, ~61.9 °, ~74.1 ° and ~78.0 °, which corresponds to the (110), (111), (200), (220), (311), (222) crystal planes of the face-centered cubic (FCC) of Cu_2_O. The XRD pattern confirms the formation of pure Cu_2_O particles and the peak indexing is in good agreement with the Joint Committee on Powder Diffraction Standards (JCPDS) file number 05-0667. The high intensity peaks indicate crystalline Cu_2_O powder. The broad XRD peaks indicate fine crystallite size. The average crystallite size of Cu_2_O particles as estimated by Scherrer’s equation was found to be ~7.68 nm. However, these fine crystallites aggregated together to form sub-micron sized particles.

Figure 1(b) elucidates the FTIR spectrum of the Cu_2_O sample. In the FTIR spectrum, the strong band at 630 cm^−1^ is assigned to the Cu (I)-O stretching vibration. The band at 1095 cm^−1^ is attributed to the presence of the oxygen bonding in copper.[17,18] The broadband at 3428 cm^−1^ is mainly due to the O-H stretching bonds of hydroxyl groups, which can be formed by the absorbed water on the surface of particles. The peaks at 1625 cm^−1^ (asymmetric vibration) and 1370 cm^−1^ (symmetric vibration) indicate the presence of the (–COO–) carboxylate ions. The occurrence of these functional groups indicates the active functionalized surfaces of the particles that are useful in the adsorption process.[17,19]

Figure 1(c) shows a UV-vis absorption spectrum of the Cu_2_O sample. The powder was first suspended in the ethanol and then exposed to ultrasonic vibration for better dispersion. The broad and strong peak at 370 nm reveals the high crystallinity of the Cu_2_O particles.[18,19] The appearance of peak at ~285 nm might be due to the orientation of the grains and surface defects, which in turn leads to the light confinement at the grain boundaries.[19]

Figure 1(d) shows the Raman spectrum of the Cu_2_O sample, revealing the characteristic phonon frequencies of the crystalline Cu_2_O particles. The strong peak at ~272 cm^−1^ is due to the second-order Raman-allowed mode of the Cu_2_O crystals. It is also an indication of oxide layer on the copper particles.[20] The intense band at 609 cm^−1^ is attributed to the infrared-allowed mode of the Cu_2_O particles.[21] The bands at 460, 609, 731, 922 and 1067 cm^−1^ (under 532 nm laser line excitation) can be assigned to the resonance with the dipole-allowed exciton states of the Cu_2_O particles.[19,22]

**Figure 2.**
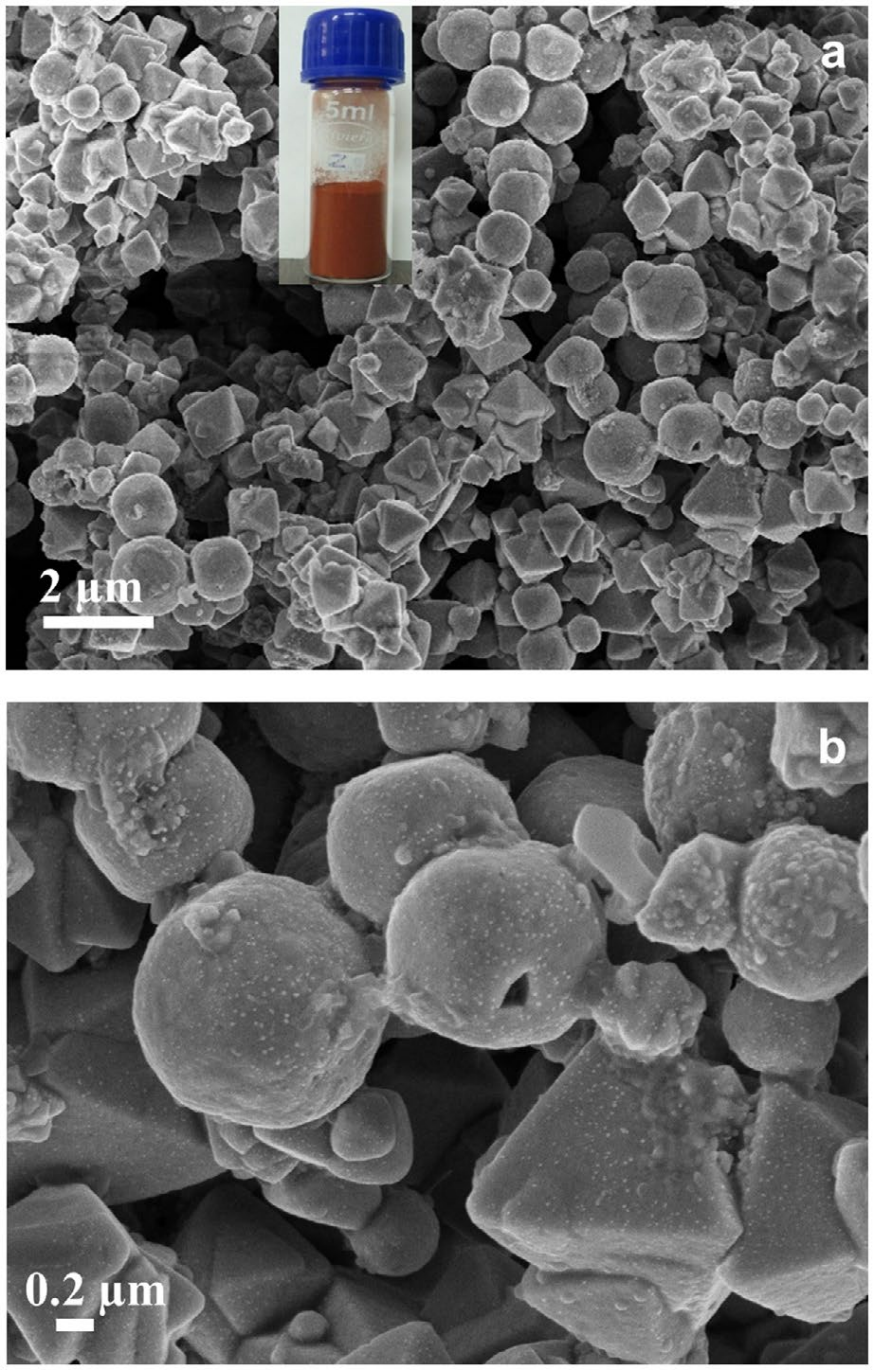
Low (a) and high (b) magnification FESEM micrographs of Cu_2_O particles. The inset is a digital photograph of the Cu_2_O powder.

Typical FESEM images of Cu_2_O particles are shown in Figure 2. These particles displayed different types of morphologies (octahedra, spheres, pyramids, cones and wires) with various sizes. However, it is observed that the spheres and pyramids are the predominant shapes. This is likely due to the unique nature of the reducing agent DFPES, which has compounds rich with hydroxyl and carboxyl groups as indicated by the FTIR spectroscopic analysis. It can be clearly observed from the micrographs that each particle is constituted by smaller features (most probably small crystallites as indicated by XRD results). Such features are attributed to the non-uniform nucleation rate and the formation of less number of nuclei in the solution. In the subsequent step, during the particles’ growth, the aggregation of new nuclei around the already formed nuclei aids the formation of large particles.[23] One solution to avoid agglomeration is to increase the concentration of DFPES, which may not only allow a uniform nucleation but also result in the formation of a large number of nuclei.

### 4.2. Reaction mechanism

The structure and the surface properties of the Cu_2_O particles have been verified in the above analysis. The major questions that are needed to be addressed in the context of reaction mechanism are: (1) which component of the DFPES is the major contributor for the reduction mechanism? And (2) what is the role of NaOH in the reaction?

To answer these questions, two new sets of experiments were carried out. In the first experiment, the graded D-glucose (Merck, 99%) of high purity was used as a reducing agent, and the results are compared with those obtained using DFPES*.* In the second set of experiments, the reaction was performed only in alkali medium by using sodium hydroxide (without DFPES or D-glucose). In the case of NaOH, the following reaction takes place:

(3) $${\text{CuCl}}_{2}\cdot 2{\text{H}}_{2}\text{O}+2\text{NaOH}\to \text{Cu}{\left(\text{OH}\right)}_{2}+2\text{NaCl}+2{\text{H}}_{2}\text{O}$$

Note that NaOH does not promote the reaction to the CuO unless other reaction parameters such as the use of organic solvents, high temperature reactions and/or addition of foreign reducing agents are used. When heated above 150 °C, copper hydroxide decomposes into a black colored cupric oxide (CuO) powder (and water vapor) according to the following equation:

(4) $$\text{Cu}{\left(\text{OH}\right)}_{2}\to \text{CuO}+{\text{H}}_{2}\text{O}$$

**Figure 3.**
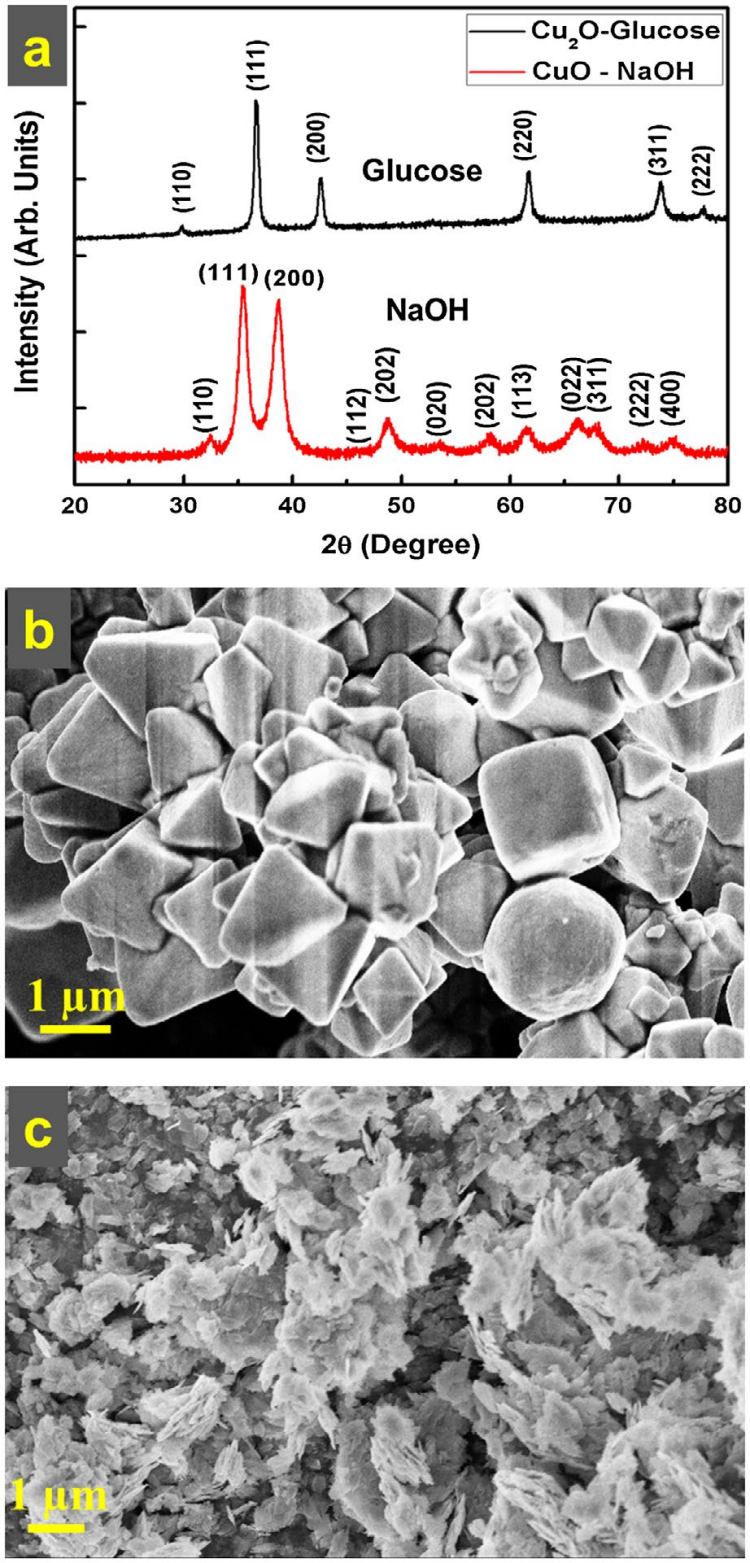
(a) XRD pattern of Cu_2_O particles prepared in aqueous solution with the aid of graded glucose and XRD pattern of CuO particles prepared with only NaOH. (b) FESEM micrograph of Cu_2_O particles prepared with the aid of graded glucose in aqueous medium and (c) FESEM micrograph of CuO particles prepared with the aid of NaOH in aqueous medium.

Figure 3(a) depicts the X-ray diffractograms of the as-prepared samples by using D-glucose and NaOH. The XRD pattern from the sample wherein D-glucose was used as the reducing agent shows the characteristic peak positions of fcc Cu_2_O as per the JCPDS file number 065-3288. The sharp peaks in the D-glucose case were compared to those in the DFPES case, indicating the formation of large crystallites in the D-glucose case. Similarly, the XRD pattern (Figure 3(a)) from the sample, wherein only NaOH was added to the precursor solution under similar experimental conditions, shows the formation of cupric oxide (CuO) instead of Cu_2_O. This clearly indicates that NaOH does not act as a reducing agent in the reaction.

Figure 3(b) and (c) show the morphology of the as-prepared samples by D-glucose and NaOH, respectively. The mixed morphologies (Figure 3(b)) of the spheres, cubes, octahedrons, and pyramids were clearly observed in the case of graded glucose, and are quite similar to those of the Cu_2_O particles obtained when DFPES was used in aqueous solution. The particles are faceted and large. Micro-flakes were observed in the case of CuO formation (as shown in Figure 3(c)) when NaOH alone was used. These micro-flakes have different characteristics than the particles obtained in the case of graded glucose or DFPES. These two experiments confirmed the following facts; as anticipated, glucose is the main reducing agent in the DFPES while NaOH has negligible reduction capability.

Based on the above presented experimental analysis, the following reaction pathways are proposed for the formation of Cu_2_O particles when DFPES is used to react with Cu salt solution in the presence of NaOH in aqueous medium:

(5) $$\text{CuC}{\text{l}}_{2}\cdot 2{\text{H}}_{2}\text{O}+2\text{NaOH}\to \text{Cu}{\left(\text{OH}\right)}_{2}\cdot 2{\text{H}}_{2}\text{O}+2\text{NaCl}$$

(6) $$2\text{Cu}{\left(\text{OH}\right)}_{2}\cdot 2{\text{H}}_{2}\text{O}+{\text{C}}_{6}{\text{H}}_{12}{\text{O}}_{6}\left(aq\right)\to \text{C}{\text{u}}_{2}{\left(\text{OH}\right)}_{2}\cdot 2{\text{H}}_{2}\text{O}+{\text{C}}_{6}{\text{H}}_{12}{\text{O}}_{7}+3{\text{H}}_{2}\text{O}$$

(7) $${\text{Cu}}_{2}{\left(\text{OH}\right)}_{2}\cdot 2{\text{H}}_{2}\text{O}\to {\text{Cu}}_{2}\text{O}+3{\text{H}}_{2}\text{O}$$

NaOH promotes the formation of copper hydroxide (Equation (5)). The reduction of copper hydroxide to Cu_2_O particles using glucose is performed in two steps. Gluconic acid (C_6_H_12_O_7_) was formed during the reaction and probably considered the stabilizing agent of Cu_2_O particles.[9,25] Glucose and NaOH drive the reactions by providing appropriate amount of hydroxyl and carboxyl ions.[8,24]

### 4.3. Effect of organic solvent on the copper precursor

**Figure 4.**
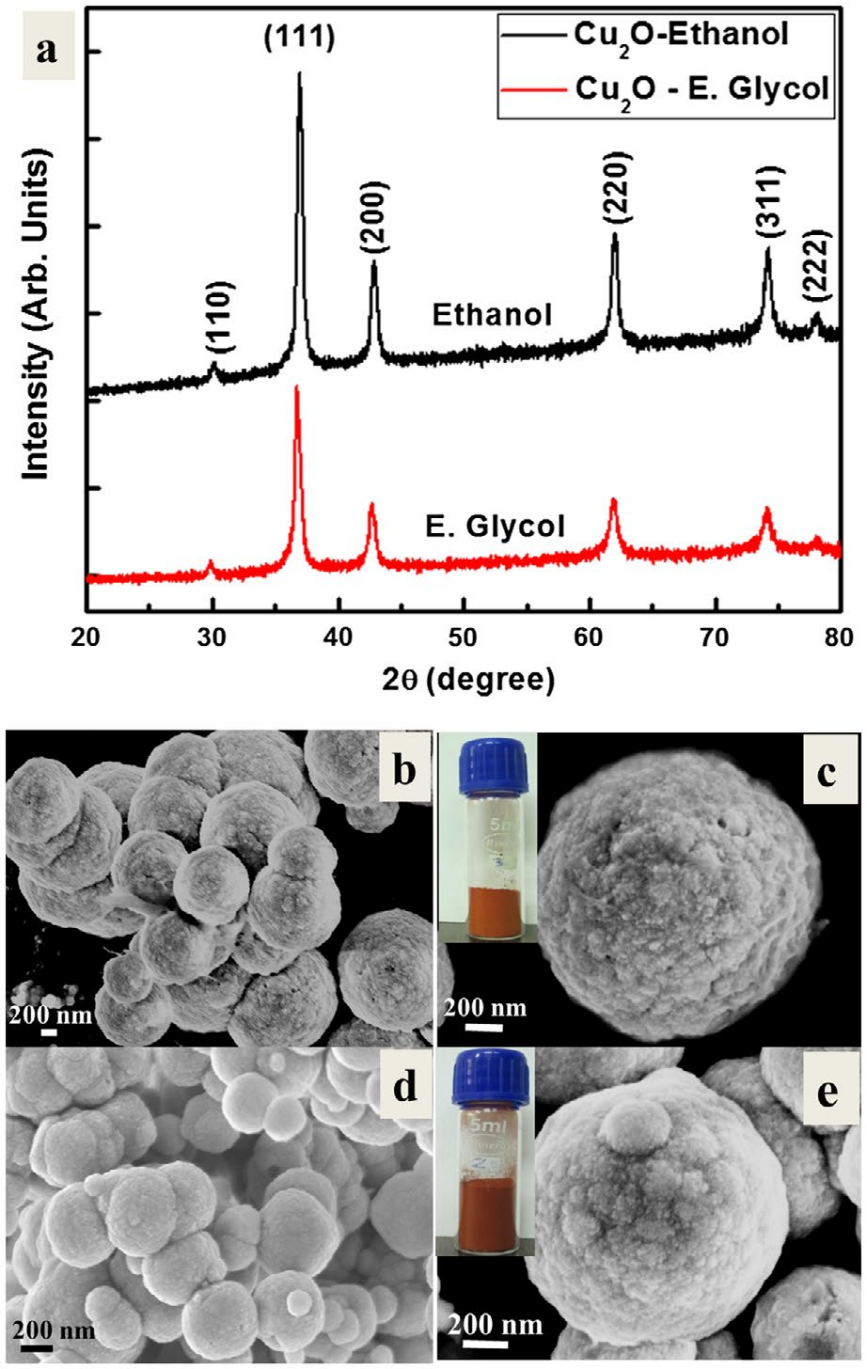
(a) XRD patterns of the Cu_2_O particles prepared by DFPES using ethanol and ethylene glycol (E. Glycol) solvents. FESEM micrographs of the Cu_2_O particles prepared by the DFPES at (b, c) ethanol and (d, e) ethylene glycol solvents. Inset figures are the corresponding Cu_2_O powders.

In this section, ethanol and ethylene glycol are used as organic solvents for the copper precursor, to investigate the effect of these solvents on the morphologies and crystallinity of the Cu_2_O particles. Figure 4(a) shows the XRD diffractograms of these samples. The diffraction peaks confirm the structure and the phase purity of the Cu_2_O particles. The samples exhibited similar crystallinity of that Cu_2_O particles obtained using water as the solvent (Figure 1(a)). However, the diffraction peaks are found to be comparatively narrow in the case of ethanol and ethylene glycol, indicating that the sizes of the Cu_2_O crystallites could be slightly larger in comparison to those obtained using water. In contrast, the diffraction peaks of Cu_2_O crystallites in the case of ethylene glycol solvent are broader than in the case of ethanol solvent, indicating that the sizes of the Cu_2_O crystallites could be slightly smaller in sizes in the case of ethylene glycol solvent.

Figure 4(b–e) show the FESEM micrographs of the Cu_2_O particles obtained using ethanol and ethylene glycol as the solvents. The reddish color (inset of Figure 4(c) and (e)) is an indication of the formation of Cu_2_O similar to the case of water as the solvent. It can be seen from the FESEM images that that spherical particles of different sizes are formed when organic solvents are used. The organic solvents have a large number of hydroxyl ions that are normally essential for oxidation reactions.[25,26] These ions uniformly occupy the surfaces of all the growing crystalline planes and hinder the growth of particles along these planes, yielding a spherical morphology.[27,28]

When metal ions are dissolved in an organic solvent (such as ethanol and ethylene glycol), the reaction conditions allow the solvent to act as a reducing agent.[27] Amongst these solvents, the relative polarity of ethylene glycol (0.79) is larger than that of ethanol (0.65), which suggests high solubility and rapid dissociation of the copper precursor in ethylene glycol than ethanol. Moreover, the lower the polarity of the solvent, the weaker is the electrostatic stabilization and dielectric constant and therefore low electron transfer rate. High magnification FESEM micrographs of the Cu_2_O particles obtained in the case of the ethanol and ethylene glycol solvents are shown in Figure 4(c) and (e), respectively. It has been observed that each particle contains a large number of fine particulates, which aggregated to form large particles. The development of the large particle is attributed to the formation of a limited number of metal nuclei in the first step, arising from low activity and poor distribution of the reducing agent’s species in the reaction system. The nuclei are then grown on account of the other generated atoms to form the primary particles. The use of strong reducing agents enables a major diffusion of the electrons homogenously and the aggregation rate of the atoms slows.[28] The use of an appropriate solvent might hinder the aggregation and accelerate the formation of uniform-sized particles.[27] In this case ethylene glycol functions as a solvent, surfactant and reducing agent. Thus, it is clear that organic solvents have an effect on the final geometry of the particles.

## 5. Removal of As(III) from water

Basically, an adsorption is a mass transfer process in which the toxic ions in a liquid are diffused to the surface of a solid adsorbent; and bound by physical and/or chemical interactions. Active functional groups with engineered surfaces are essential for good adsorption properties.[3,29] Herein, the synthesized Cu_2_O particles in aqueous medium were used as an adsorbent to uptake the toxic As(III) ions from the water. As it is cost effective, non-toxic, and has negligible solubility in water, the use of Cu_2_O powder as an adsorbent is highly desirable. In this section, kinetic and batch studies were carried out to investigate the adsorption properties of this material. Adsorption isotherms and adsorption kinetic models were used to estimate the adsorption capacity and to identify the adsorption mechanism.

### 5.1. Batch study

**Figure 5.**
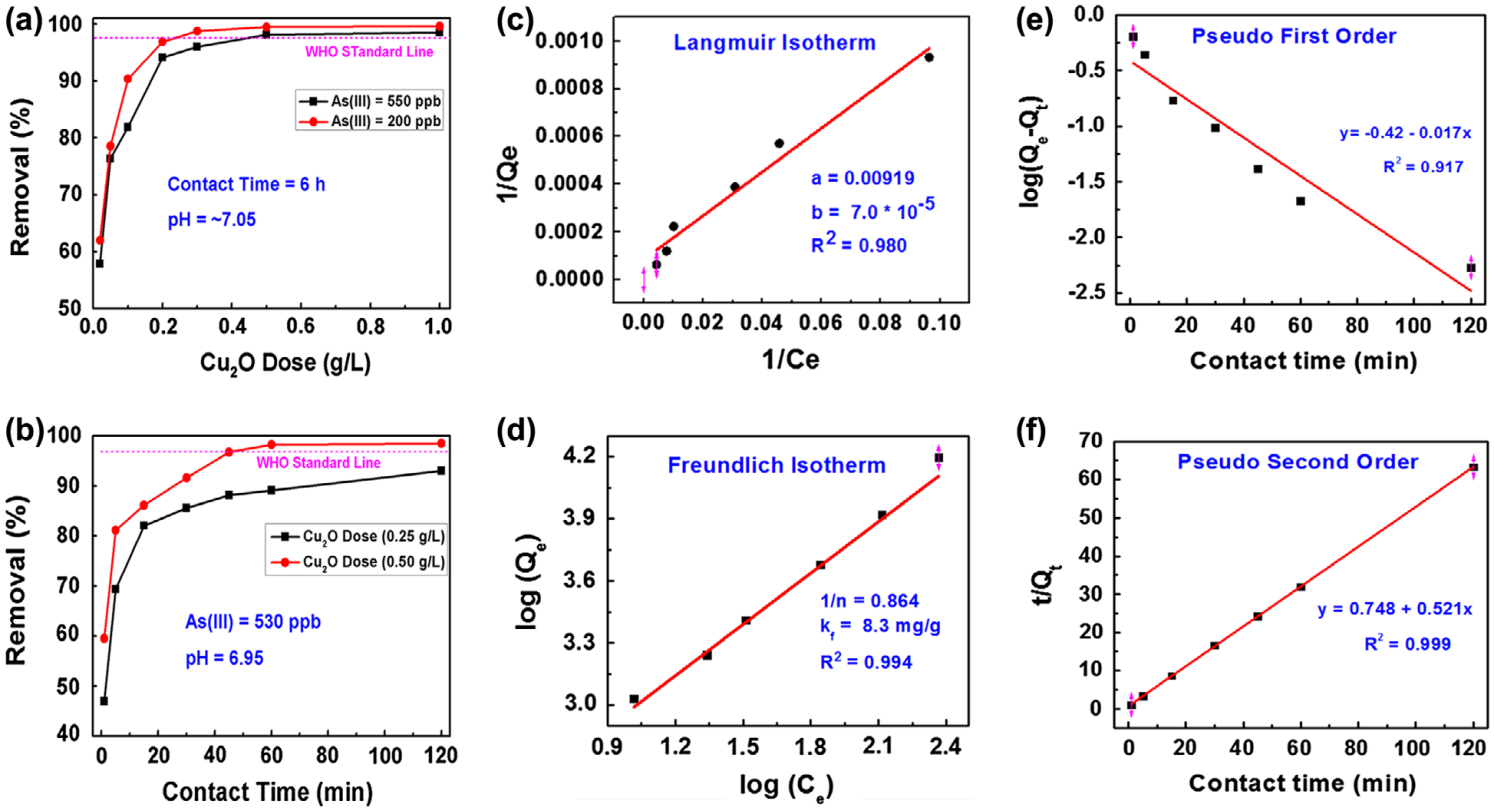
(a) Adsorption dose and (b) adsorption time as a function of As(III) removal (%) by Cu_2_O adsorbent; (c) Langmuir and (d) Freundlich adsorption isotherms; (e) pseudo-first order and (f) pseudo-second order adsorption kinetics data. WHO standard line is included for reference in (a) and (b).

Figure 5(a) shows the results of the adsorption batch study corresponding to Cu_2_O adsorbent and As(III) removal efficiency. It was performed to estimate the optimum Cu_2_O dose required to attain the equilibrium state. Two parallel experiments at initial As(III) concentrations of 200 and 550 ppb were performed in this study. Seven doses of the Cu_2_O adsorbent were chosen from 0.02 to 1.0 g per liter of the As(III) solution. It was found that the removal efficiency increases with the adsorbent dosage up to a certain limit and then attained saturation. The results infer that As(III) removal efficiency has increased from 57.6% to 99.2% as the adsorbent dose increased from 0.02 to 1 g l^–1^. The optimum adsorbent doses corresponding to the As(III) 550 ppb and As(III) 200 ppb concentrations were found to be 0.5 and 0.2 g l^–1^, respectively. The removal efficiency of Cu_2_O adsorbent is better than that reported for CuO adsorbent for arsenic removal.[14–16]

### 5.2. Kinetic study

Figure 5(b) shows the graph of the contact time (pertaining to Cu_2_O adsorbent) on the *x-axis* and As(III) removal efficiency (removal (%)) on the *y*-axis. The contact time required to completely adsorb the As(III) ions from the water onto the surface of the Cu_2_O particles is also an important factor when considering the adsorbent’s practical feasibility. To study this effect, two experiments were carried out at 0.25 and 0.50 g l^–1^ adsorbent doses. The As(III) concentration and pH of the solution were fixed at 530 ppb and 6.95, respectively, and the experiments were conducted at different contact times (1–120 min). It was found that with 0.25 g l^–1^ adsorbent dose, more than 80% of As (III) is removed in the first 20 min, and afterwards the adsorption slows down before reaching equilibrium. However, with 0.50 g l^–1^ adsorbent dose, 98.25% of As(III) ions were adsorbed within the first hour. This is the safe limit of As(III) in drinking water (the WHO limit for As(III) in drinking water is <10 ppb). These observations indicate fast and efficient removal of As(III) with minimum amount of Cu_2_O adsorbent than the reported removal efficiencies of copper oxides in the literature. [14–16]

### 5.3. Adsorption isotherms

Figure 5(c) shows the Langmuir adsorption isotherm plotted using the data depicted from dose study at the As(III) concentration of 550 ppb as a reference datum. The maximum adsorption capacity is a leading parameter in the adsorption process and can be estimated using adsorption isotherms such as Langmuir adsorption model,[30] at constant temperatures according to Equations (8 and 9).

(8) $$\frac{1}{Q_{e}}=\frac{1}{Q_{\max}}+\frac{1}{K_{L}\cdot Q_{\max}}\left(\frac{1}{C_{e}}\right)$$

The Langmuir adsorption isotherm was plotted with the *x*-axis as (1/*C*_*e*_) and the *y*-axis as (1/*Q*_*e*_). The parameters *a* = 1/(*K*_*L*_ *. Q*_max_) and *b* = 1/*Q*_max_ are determined from the Langmuir graph as shown in Figure 5(c). The value of *Q*_max_ is calculated from the parameter *b* in which *Q*_max_ = 1/*b* = 14.285 mg g^–1^ and the Langmuir constant, *K*_*L*_ = 0.0076 l μg^–1^.

The equilibrium parameter (*R*_*L*_) is a dimensionless parameter, which is mainly used to determine the applicability of an adsorbent for the removal of an adsorbate in aqueous solution. The value of *R*_*L*_ can be defined as:

(9) $$R_{L}=\frac{1}{1+K_{L}\cdot C_{\text{0}}}$$

The values of *C*_0_ (initial As(III) concentration) = 550 μg l^–1^ and *K*_*L*_ = 0.0076 l μg^–1^ and therefore, *R*_*L*_ = 0.1942, which is a favorable value for the adsorption process.

Figure 5(d) shows the Freundlich adsorption isotherm, which is an optimum way to describe the adsorption mechanism of heterogeneous surfaces. The equations represent the Freundlich adsorption isotherm [31] and its linear form are as follows:

(10) $$Q_{e}=K_{f}C_{e}^{\frac{1}{n}}$$

(11) $$\log\left(Q_{e}\right)=\log\left(K_{f}\right)+\frac{1}{n}\log\left(C_{e}\right)$$

where *K*_*f*_ is the Freundlich constant (mg g^–1^) and *n* is adsorption intensity. Freundlich isotherm is plotted in a linear algorithmic plot based on Equation (11) where the *x*-axis is log(C_e_) and the *y*-axis is log(Q_e_). The parameters *K*_*f*_ and 1/*n* are determined from the intercept and slope of the plots of the Freundlich graph. The value of ‘1/*n*’ is another indication to the enhanced adsorption characteristics of the Cu_2_O adsorbent. In the present analysis, it is found that 1/*n* = 0.864, which indicates favorable adsorption characteristics of the Cu_2_O adsorbent.[29] Similarly, log(*K*_*f*_) found to be 2.113, and *K*_*f*_ is 8.3 mg g^–1^, which is the specific adsorption capacity of the Cu_2_O adsorbent.

### 5.4. Adsorption kinetics

The adsorption kinetics data is presented in Figure 5(e) and (f) as fitted to pseudo first order and pseudo second order Equations (12) and (13), respectively. Pseudo first order kinetic model is represented by linear Lagergren equation [32] as follows:

(12) $$\log\left(Q_{e}-Q_{t}\right)=\log\left(Q_{e}\right)-\frac{K_{1}\cdot t}{2.301}$$

where *Q*_*t*_ is the amount of adsorbate adsorbed at time *t* measured in mg g^–1^. *K*_1_ is equilibrium rate constant of pseudo first order adsorption. Figure 5(e) indicates poor fitting (low regression coefficient (*R* ^2^)) of the data with pseudo first order model, which indicates that the adsorption is not physisorption. However, by considering the points of the first hour contact time only, the points are fitted well with pseudo-first order adsorption. Pseudo second order model is a nonlinear model that describes the surface adsorption in terms of physicochemical interaction between adsorbate ions (As(III)) and the adsorbent surfaces (Cu_2_O). It is usually plotted based on the following Equation (13):

(13) $$\frac{t}{Q_{t}}=\frac{1}{k_{2}\cdot Q_{e}^{2}}+\frac{1}{Q_{e}}t$$

where *K*_2_ and *Q*_*e*_ can be determined from the intercept and slope of *t/Q*_*t*_ vs*. t* plot, respectively. *K*_2_ is a pseudo-second order model constant that describes the adsorption rate and is measured in g mg^–1^ min^–1^. Figure 5(f) indicates excellent fitting (very high *R* ^2^) of the data with pseudo-second order model, which indicates that pseudo-second order model (indicating chemical nature of the adsorption) is the appropriate model to identify the adsorption kinetics of As(III) ions onto Cu_2_O surfaces. The above experimental observations clearly indicate the following findings: (I) adsorption in the first hour contact time is physicochemical in nature but by considering the whole range of the contact time the adsorption is chemisorption; [33–35] (II) the adsorption mechanism depends mainly on the availability of adsorption sites on the surface of Cu_2_O adsorbent rather than the concentration of As(III) in water;[35] and (III) the adsorption data can be well fitted by both Langmuir and Freundlich isotherms indicating the complexity of the adsorption process.[36]

## 6. Conclusions

The present study demonstrated an easy single-step green synthesis process of polycrystalline Cu_2_O particles with controlled morphologies. The chemical reaction is carried out in aqueous medium with the aid of extract from date fruit pulp and without using any toxic chemicals. The results revealed that the high glucose content in the date fruit pulp acts as a reducing agent in the formation of Cu_2_O particles, thus eliminating the use of expensive pure sugar alternatives. The use of organic solvents such as ethanol and ethylene glycol resulted in the formation of spherical particles while the use of water as a solvent resulted in mixed morphologies. The synthesis protocol used in this work allows upscaling owing to its innate nature. Fourier transform infrared spectroscopy analysis of the Cu_2_O particles showed that the surfaces of the particles were functionalized with hydroxyl and carboxyl groups, which were found extremely useful in the effective uptake of As(III) ions by Cu_2_O particles from the water. The adsorption kinetic and batch studies showed excellent adsorption characteristics of the Cu_2_O particles. The adsorption capacity as estimated by Langmuir adsorption isotherm was ~14.3 mg g^–1^. Adsorption kinetics followed a pseudo-second order model which indicated that the adsorption is chemisorption.

## Disclosure statement

No potential conflict of interest was reported by the authors.

## Funding

This work was supported by deanship of scientific research at King Saud University, Research Group Program [grant number RGP-1453-078], and The Science & Engineering Research Board (SERB), Department of Science and Technology (DST), India [grant number SERB/F/3487/2012-2013].
